# High prevalence but low concentrations of blood lead (Pb) levels among trumpeter swans in central North America

**DOI:** 10.1007/s10646-026-03054-3

**Published:** 2026-02-21

**Authors:** David W. Wolfson, John R. Fieberg, David E. Andersen

**Affiliations:** 1 Department of Fisheries, Wildlife, and Conservation Biology, Minnesota Cooperative Fish and Wildlife Research Unit, University of Minnesota, Saint Paul, MN 55108 USA; 2 Department of Fisheries, Wildlife, and Conservation Biology, University of Minnesota, Saint Paul, MN 55108 USA

**Keywords:** Biosentinel, Cygnus buccinator, Lead, Trumpeter swan, Waterfowl

## Abstract

Lead (Pb) is a toxic heavy metal that has detrimental effects on wildlife. The toxicant is particularly relevant for species that use a muscular gizzard to digest food and that forage in areas with accumulated sediment at the bottom of lakes and streams. Poisoning from ingested spent lead ammunition or fishing tackle has been a major source of mortality of Trumpeter Swans (*Cygnus buccinator*) across North America. However, virtually all lead testing has been conducted on carcasses or swans brought to wildlife rehabilitation centers, and there are no baseline data from free-ranging swans. We estimated blood lead concentrations for 115 free-flying Trumpeter Swans throughout the breeding range of the Interior Population that inhabits central North America. We detected lead in all samples, but 90% of swans had blood lead levels in the ‘background’ range (< 200 ppb), 8% of swans had blood lead levels in the ‘sub-clinical’ range (200–500 ppb), and 2% of swans had blood lead levels in the ‘clinical’ (500–1,000 ppb) or ‘severe’ (> 1,000 ppb) range of lead toxicity. The swan with the highest blood lead level (1,076 ppb) was captured 3 km from a major coal power plant, although we were unable to definitively trace the source of lead (e.g., to coal ash or fishing tackle) in that individual. Our study provides useful baseline information on the prevalence and magnitude of blood lead levels among Interior Population trumpeter swans and demonstrates the apparent continued wide-scale exposure of wetland-feeding waterfowl to lead.

**Supplementary Information:**

The online version contains supplementary material available at 10.1007/s10646-026-03054-3.

## Introduction

Lead, a toxic heavy metal with detrimental effects on wildlife, is pervasive throughout the environment due to anthropogenic activity (Pain 1996; Saaristo et al. 2018; Pain et al. 2019). Lead disrupts multiple molecular pathways (Mitra et al. 2017), and absorption of even trace amounts of lead can result in a variety of sublethal effects to health, reproduction, and behavior; higher quantities can result in mortality of many species of birds (Fisher et al. 2006; Monclús et al. 2020). Exposure to lead has been linked to reductions in long-term population growth rates, though the impacts of lead differ among demographic categories (e.g., age, sex, breeding status; Sanderson and Bellrose 1986; Mateo 2009; Flint et al. 2016; Pain et al. 2019).

Lead poisoning is a particularly harmful threat for waterfowl species that consume grit to facilitate breaking down food during digestion (Sanderson and Bellrose 1986; Pain et al. 2019). Ingested lead, consumed while foraging, is retained in the gizzard until it is broken down by the muscular grinding action and the acidic conditions (pH 2.5) in the stomach, forming soluble lead salts that are absorbed into the bloodstream and can bioaccumulate, particularly in the liver, kidney, brain, and bone (Pain 1996; Cheek and Marteel-Parrish 2013; Pain et al. 2019).

There are two primary sources of lead to which waterfowl are exposed: spent lead ammunition and fishing tackle (Rattner et al. 2008). Within the USA, lead exposure from shot used in waterfowl hunting has markedly declined since the nationwide ban on use of lead ammunition in aquatic habitats in 1991. However, lead from historical use of toxic ammunition continues to be a threat to waterfowl populations because it persists in sediments and may become periodically available depending upon environmental conditions (e.g., drought or draw-down conditions that increase availability; Anderson et al. 2000; Samuel and Bowers 2000; Flint and Schamber 2010; Check and Marteel-Parrish 2013). Lead concentrations in waterfowl populations tend to decline following the banning of lead shot in wetlands. However, individuals sampled decades after the lead ammunition ban still test positive for the presence of lead (Lewis et al. 2021), and evidence suggests that waterfowl still ingest remnant spent ammunition in the sediment of wetlands (Anderson et al. 2000).

Most understanding of lead levels in wildlife comes from animals with lead poisoning that are brought into wildlife rehabilitation facilities. Much less is known about the levels of lead present in wild, free-ranging animals, the overall prevalence of bioavailable lead on the landscape, and the degree to which avian populations can tolerate lead exposure without major deleterious effects (Monclús et al. 2020; Cox 2021). Critical knowledge gaps remain in understanding how subclinical (not producing apparent symptoms) and sublethal lead exposure affect behavior and physiology of large waterfowl (Haig et al. 2014; Kearns et al. 2019). Subclinical levels of lead exposure do not cause outwardly conspicuous effects, but can produce negative physiological symptoms such as the inhibition of $\:\delta\:$-aminolevulinic acid dehydratase (ALAD), an essential enzyme needed for red blood cell production (Hoffman et al. 1981; Pain 1989). Clinical levels cause more externally detectable signs, such as weakness, weight loss, regurgitation, loss of muscular coordination, change in vocalization, and abnormal feces (Routh and Sanderson 2009; Franson and Pain 2011).

Swans (*Cygnus* spp.) are especially susceptible to lead ingestion because their long necks allow them to forage for sub-emergent vegetation and grit in the substrate of shallow wetlands, thereby enabling them to potentially ingest lead shot and fishing tackle that accumulate in the sediment layer (Banko 1960; Blus et al. 1989; Degernes et al. 2002). The longevity of lead in wetland systems means that even historical accumulation of lead still poses a threat to swans. Thus, today, lead poisoning is a major source of mortality, especially when swans concentrate in areas with historical lead accumulation during the non-breeding period (Blus 1994; Lagerquist et al. 1994; Degernes et al. 2006). The risk of exposure is particularly exacerbated during periods of drought, when low water levels allow waterfowl to reach lake sediment that is otherwise inaccessible. Drought conditions resulted in a large-scale lead-induced mortality event in Washington State involving > 100 individual swans (Wilson et al. 1998). Wilson et al. (2009) found that lead shot was the source of > 80% of 2,577 swan mortalities in northwestern Washington and southwestern British Columbia during the winters of 1999–2008, although their sample consisted entirely of opportunistically recovered carcasses.

Trumpeter swans (*Cygnus buccinator*) were extirpated from the "Interior Population" (IP) in central and eastern North America during the 19th century due to unregulated market hunting (Banko 1960). Reintroductions to this historical breeding range began in the 1960s, and swans in the IP currently breed throughout the western Great Lakes region of the U.S. and Canada (Groves 2017). Although multiple studies have quantified lead levels associated with IP trumpeter swan carcasses, sufficient comparable data are not available for live IP swans (Strom et al. 2009; Degernes and Frank 2013; Cox 2021).

Our main objective was to quantify concentrations of lead in trumpeter swans across the IP breeding range to provide a contemporary summary of the overall prevalence and magnitude of blood lead levels among wild IP trumpeter swans > 40 years following banning of lead shot for waterfowl hunting in most of North America. We aimed to address the following questions: (1) what are the blood lead concentrations in wild-ranging IP trumpeter swans, and (2) how do these levels vary with age, sex, and body size? We predicted that (1) given the pervasiveness and persistence of lead across the landscape, prevalence of lead in trumpeter swans is likely high, and (2) lead concentrations would be higher in adults than young because there are opportunities for lead exposure in each year of an animal's life.

## Methods

### Study site and sample collection

We captured trumpeter swans across the breeding range of the IP, including the main contiguous Great Lakes areas and the ‘High Plains’ flock in western Nebraska (Groves 2017) from July 2019 through August 2022 (Wolfson 2024; Wolfson et al. 2024). We used a variety of watercraft to capture swans, including jon boats with long-tail mud motors, airboats, and step deck transom boats with surface-drive motors. Upon approaching a molting flightless swan via watercraft, we hand-captured it using a shepherd’s crook pole (Eltringham 1978; Hindman et al. 2016). We manually restrained captured swans and banded them with U.S. Geological Survey leg bands. We determined sex via cloacal examination and age (e.g., either adult or cygnet) via plumage characteristics. We measured body mass using a spring scale (± 0.1 kg) and tarsus length using a sliding caliper (± 1.0 mm; measured from the notch of the intertarsal join to the end of the tarsus bone). We also affixed a GPS-GSM tracking collar to a subset of captured swans as part of a study assessing their movement. We collected 2 mL of whole blood from each captured swan via medial metatarsal venipuncture using a 23-gauge needle with a 3-mL syringe, stored samples in 3-mL K2 ethylenediaminetetraacetic acid (EDTA) additive lavender-top venous blood collection serum tubes (Vacuette brand, Greiner Bio-One, Monroe, North Carolina, USA) to prevent coagulation, and refrigerated tubes until shipping them to a diagnostics lab. Protocols for capturing and drawing blood from Trumpeter Swans were approved by the University of Minnesota Animal Care and Use Committee (protocol no. 1905-37072A), the U.S. Geological Survey Bird Banding Laboratory (Federal Bird Banding Permit no 21631), and state-specific permit approved by each state wildlife agency involved.

### Laboratory analysis

Lab technicians at the Iowa State University Veterinary Diagnostic Laboratory (ISU-VDL) used Inductively Coupled Plasma Mass Spectrometry (ICP-MS) according to internal standard operating procedures to quantify blood lead concentrations in parts per billion (ppb). Analysis was performed with a Plasma Quant Elite ICP-MS (Analytik Jena Inc. Woburn, MA, USA) in CRI mode with hydrogen as the skimmer gas and bismuth as the internal standard. Standards for elemental analyses were obtained from Inorganic Ventures (Christiansburg, VA), and 15 mL centrifuge tubes and trace mineral grade nitric acid were obtained from Fisher Scientific (Pittsburgh, PA, USA). Samples were digested in concentrated nitric acid by adding 0.25 mL of acid to 0.25 mL of blood at 70 °C for one hour in a 15 mL centrifuge tube. After digestion, the digest was brought to 5 mL with 18 MOhm deionized water, mixed and centrifuged. Sample digests were filtered through a $\:0.45\mu\:m$ syringe filter. All samples were rigorously mixed, then analyzed by ICP-MS. For quality control, a control blood sample was analyzed with each analytical run and determined to be within 2 standard deviations of the expected concentration. The limit of detection (i.e., the lowest possible concentration at which the method can detect lead; LOD) using the equipment at ISU-VDL is 0.054 ppb, and the limit of quantification (i.e., the lowest possible concentration of lead that can be reliably detected and quantified; LOQ) is 2.0 ppb (Armbruster and Pry 2008). To compare results with other studies of blood lead concentrations reporting in $\:\mu\:g/dL$, 10 ppb is equivalent to 1 $\:\mu\:g/dL$.

We assessed blood lead concentrations according to the following categories from Pain (1996) and Franson and Pain (2011): <200 ppb is considered ‘Background’ level (also referred to as normal environmental exposure), 200–500 ppb is considered ’Subclinical’ lead poisoning, 500–1000 ppb is considered ‘Clinical’ lead poisoning, and > 1000 ppb is considered ‘Severe’ clinical lead poisoning.

### Statistical analysis

We modeled the relationship between blood lead concentrations and covariates (age, mass, sex, and tarsus) using a generalized linear model with a Gamma error distribution and a log link function. We included age class (e.g., adult, cygnet) as a covariate because we expected lead concentrations would vary between age classes due to differential exposure pathways to lead. We included mass and tarsus length as potential proxies for lifespan and body condition because we expected older, healthier individuals to have higher mass and longer tarsus length. The inclusion of sex was exploratory, but we were interested in sex-specific differences in activity during nest incubation that may influence the ingestion of lead, particularly because of the potential to transfer lead from bones during the egg-laying process (Vallverdú-Coll et al. 2015). We assessed model assumptions by examining qqplots of residuals and plots of residuals versus fitted values.

We used empirical cumulative distribution functions (ECDF) to describe the cumulative proportion of swans sampled with blood lead concentrations less than or equal to a given value *x* (in ppb). We created separate ECDFs for the 2 age classes to evaluate age-related differences in exposure using the stat-ecdf function in the ggplot2 R package (Wickham 2016). The ECDFs are defined as

$$\:{\boldsymbol{F}}_{\boldsymbol{g}}\left(\boldsymbol{x}\right)=\left(1/{\boldsymbol{n}}_{\boldsymbol{g}}\right){\sum\:}_{\boldsymbol{i}=1}^{{\boldsymbol{n}}_{\boldsymbol{g}}}\boldsymbol{I}\left({\boldsymbol{x}}_{\boldsymbol{i},\boldsymbol{g}}\le\:\boldsymbol{x}\right),\hspace{1em}\boldsymbol{g}\in\:\boldsymbol{A}\boldsymbol{d}\boldsymbol{u}\boldsymbol{l}\boldsymbol{t}\boldsymbol{s},\boldsymbol{C}\boldsymbol{y}\boldsymbol{g}\boldsymbol{n}\boldsymbol{e}\boldsymbol{t}\boldsymbol{s}.$$

where $\:{n}_{g}$ is the number of swans sampled in age class $\:g$, $\:{x}_{i,g}\:$is the blood lead concentration (ppb) of the *i*th swan in that age class, and $\:I\left({x}_{i,g}\le\:x\right)$ is an indicator function taking the value 1 if the condition is true and 0 otherwise. The ECDFs provides a nonparametric summary of the observed distribution of blood lead concentrations within each age class.

## Results

**Fig. 1:**
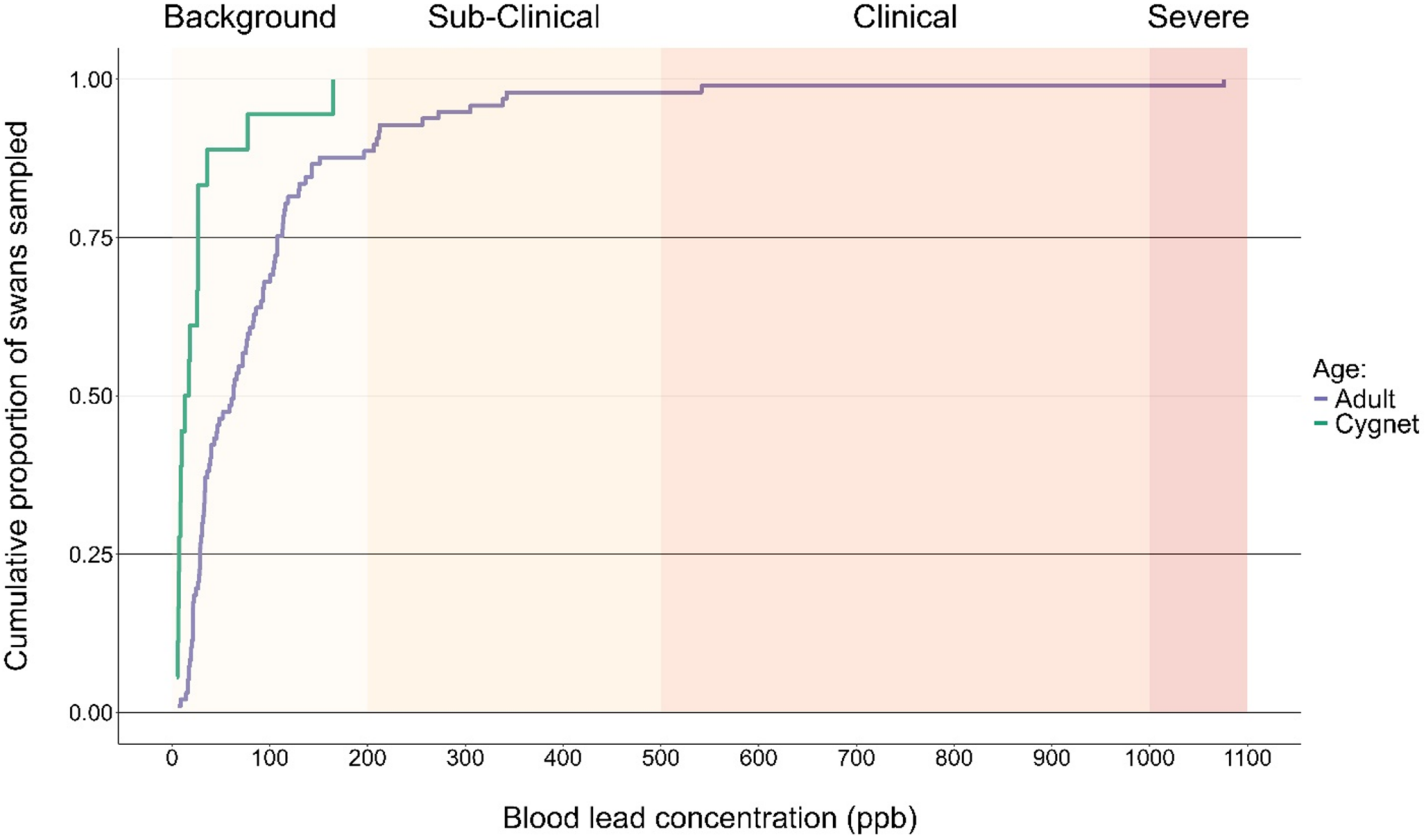
Empirical cumulative distribution function for the blood lead concentrations for 115 Interior Population trumpeter swans (97 adults and 18 cygnets) from July 2019 to August 2022 in central North America. The y-axis represents the proportion of the total sample for each group (e.g., adults and cygnets) below any given threshold of blood lead concentration in parts per billion (ppb)

We collected whole blood samples from 115 swans (97 adults and 18 first-year ‘cygnets’) during the prebasic summer molt when adult swans are flightless and cygnets have not fledged. All 115 swans tested positive (i.e., above the LOD) for lead, and all concentration values were above the reported LOQ. 90% of swans sampled (*n* = 104) had lead concentrations within the ‘Background’ category (range [4.3 ppb–196 ppb]). 8% of swans sampled (*n* = 9) had lead concentrations within the ‘Sub-Clinical’ category (range [207 ppb–342 ppb]). There was one swan in each of the highest categories of ‘Clinical’ and ‘Severe’, with lead concentrations of 542 ppb and 1,076 ppb, respectively (Fig. 1).

We did not detect significant associations between blood lead levels and mass, sex, or tarsus length (Table 1). Blood lead levels did vary significantly by age class (e.g., adult versus cygnet; *p* = 0.009), with adult swans having $\:{e}^{1.818}$= 6.12 [95% CI = 1.28–21.67] times higher blood lead concentrations, on average compared to cygnets (Table 1).

**Table 1:** Model output from a generalized linear model with a gamma error distribution and a log link function

	Coefficient	95% Confidence Interval	*p*-value
Intercept	1.748	[-2.078, 5.571]	0.365
Age	-1.818	[-3.076, -0.245]	0.009
Mass	0.024	[-0.198, 0.247]	0.813
Sex	-0.335	[-0.933, 0.274]	0.273
Tarsus	0.021	[-0.006, 0.049]	0.116

Blood lead concentrations in parts per billion from 115 Interior Population trumpeter swans were modeled as the response and predictors included age (adult is the reference level), mass (in kg), sex (female is the reference level), and Tarsus length (in mm)

## Discussion

We detected lead in every swan sampled, but most had lead concentrations that were within the background exposure range. Although lead concentrations within this range are considered below the threshold for severe effects, chronic exposure to low levels of lead may cause negative physiological effects, such as changes in behavior, physical growth, and brain development (Hoffman et al. 2000; Buekers et al. 2009; van den Heever et al. 2024). Additionally, Pain (1996) suggested that any concentration of lead will cause negative health impacts, and levels of heme synthetase and protoporphyrin IX (crucial elements in the pathway for oxygen transport in hemoglobin) may be disrupted at blood lead levels as low as 50 ppb.

It can be challenging to link blood lead levels to the environments in which exposure took place. Although often retained in the gizzard for up to six weeks, lead shot can also be evacuated soon after ingestion, which may result in minimal effects on blood lead levels (Pain 1996). Anders et al. (1982) calculated that the half-life of blood lead is 13 days in pigeons (*Columba livia*), and Fry and Maurer (2003) also estimated a half-life of 13 days for blood lead in California condors (*Gymogyps californianus*), but rigorous quantitative estimates for waterfowl species are lacking (but see Dieter and Finley 1979). Lead concentrations in our study were all from flightless swans and most likely reflect lead that swans have ingested at or near the capture area or lead ingested previously, stored in bone, and recently mobilized. Additionally, newly hatched birds may be exposed to lead from eggshells, and it is unknown what effect that may have on their lead concentrations (Vallverdú-Coll et al. 2015; Sriram et al. 2022).

No prior studies have quantified blood lead concentrations of wild trumpeter swans in the IP, and our estimates of lead concentrations establish a baseline for future research. Although other studies have quantified blood lead concentrations for trumpeter swans, their samples came from either necropsies or swans brought into wildlife rehabilitation facilities already exhibiting signs of lead poisoning, and therefore are not necessarily indicative of free-ranging wild populations (Blus et al. 1989; Katavolos et al. 2007; Strom et al. 2009; Degernes and Frank 2013). Newth et al. (2016) quantified blood lead levels in free-ranging whooper swans (*Cygnus cygnus*) in northwest England from 2010 to 2014 and found high prevalence (i.e., all swans tested positive for lead) and a higher proportion of the population above background levels, with 42% of 260 whooper swans having blood lead levels > 200 ppb. Our findings were consistent with Ely and Franson (2014), the only other study reporting the lead concentrations of a wild swan species in North America. They sampled 653 flightless tundra swans on their breeding range in northern Alaska from 2007 to 2008 and found that lead was prevalent in their sample but predominately at background levels < 200 ppb (only 5 of 653 tundra swans had subclinical levels between 200 and 500ppb, and no swans had lead levels > 500ppb consistent with clinical poisoning). Blood lead levels did not significantly vary by sex or year, but adult tundra swans had higher levels than cygnets. Thus, Ely and Fransom (2014) speculated swans were likely exposed to lead on wintering areas or on return migration to Alaska, rather than on the summer breeding grounds. However, the exact source of lead ingestion is hard to verify, because lead present in bone may be released into the blood stream during periods of bone demineralization, which can include egg laying (De Francisco et al. 2003).

Our results also highlight the potential for wildlife to serve as bioindicators across the landscape (Burger 2006a; Kays et al. 2015). For example, the swan with the highest lead level in our study (1,076 ppb), which may be indicative of recent lead ingestion, was captured ~ 3.5 km west of a large coal-fired power plant in Cohasset, MN, USA. After completing molt and replacement of its primaries, it repeatedly made trips from its breeding territory to open waste ponds at the power plant. The breeding site of that bird, Little White Oak Lake, also has a long history of fishing and waterfowl hunting, so it is likely that lead is present in the accumulated lake sediment. Despite the relatively high blood lead levels at the time of capture, that bird did not display visible signs of lead poisoning, did not die from that exposure, was observed with cygnets each subsequent year of the study (2020–2024), and migrated > 800 km to southwestern Michigan each winter.

If tracing the source point of lead is a research priority, stable isotope analysis allows researchers to differentiate types of lead based on their unique isotope ratios (Rabinowitz and Wetherill 1972). Analysis of stable lead isotopes in wild birds has been used to compare isotopic signatures from spent ammunition as opposed to either environmental pollution due to combustion of leaded gasoline or toxic waste from mining operations (Scheuhammer and Templeton 1998; Meharg et al. 2002; Scheuhammer et al. 2003). Recent work from Wang et al. (2019) demonstrated that lead isotopes in coal fly ash can be differentiated from other major anthropogenic lead sources in the United States. Thus, isotopic analysis may provide a potential tool to help identify the source of lead exposure in trumpeter swans.

Long-term monitoring of biological populations can provide the opportunity to quantify trends over time in the prevalence and bioaccumulation of heavy metals and other harmful toxicants (Golden and Rattner 2003; Burger 2006b). Avian populations across a number of different ecosystems (e.g., urban systems, wetlands, marine systems) have been used as heavy metal bioindicators (Burger and Gochfeld 2004; Zhang and Ma 2011; Cai and Calisi 2016). In addition to bone and tissues (e.g., blood, liver, kidney), heavy metal concentrations can be detected from feathers, eggs, and excrement of birds, allowing non-invasive sampling options that don’t require capture of live birds (Denneman and Douben 1993; Berglund et al. 2011; Rave et al. 2014; Hashmi et al. 2015). Collection of feathers during the prebasic molt period may provide the cheapest and least invasive sampling option, linking swans to the heavy metals present at the collection site.

Trumpeter swans in the IP are rapidly expanding in both abundance and distribution (Groves 2017). Baseline monitoring data on the overall prevalence and magnitude of lead toxicity provides useful information on the relative threat that lead poses to this population and provides an opportunity to track trends in blood lead levels over time. In addition, our results suggest that exposure to lead in trumpeter swans and perhaps other waterfowl is still widespread across central North America > 40 years following the ban on using lead shot for waterfowl hunting.

## Supplementary Information

Below is the link to the electronic supplementary material.

Supplementary Material 1

## Data Availability

Data is provided as a supplemental file.
